# SARS-CoV-2 Spike Alterations Enhance Pseudoparticle Titers and Replication-Competent VSV-SARS-CoV-2 Virus

**DOI:** 10.3390/v12121465

**Published:** 2020-12-18

**Authors:** Katherine Elizabeth Havranek, Ariana R. Jimenez, Marissa Danielle Acciani, Maria Fernanda Lay Mendoza, Judith Mary Reyes Ballista, Darren Austin Diaz, Melinda Ann Brindley

**Affiliations:** 1 Department of Infectious Diseases, College of Veterinary Medicine, University of Georgia, Athens, GA 30602, USA; katherine.havranek@uga.edu (K.E.H.); ariana.jimenez25@uga.edu (A.R.J.); marissa.acciani@uga.edu (M.D.A.); mfl71444@uga.edu (M.F.L.M.); jreyesb@uga.edu (J.M.R.B.); davdiaz01@uga.edu (D.A.D.); 2 Department of Population Health, College of Veterinary Medicine, University of Georgia, Athens, GA 30602, USA

**Keywords:** SARS-CoV-2, spike, fusion, recombinant VSV

## Abstract

Severe acute respiratory syndrome coronavirus 2 (SARS-CoV-2) is the causative agent of the most recent global pandemic that has caused more than a million deaths around the world. The spike glycoprotein (S) drives the entry and fusion of this virus and is the main determinant of cell tropism. To explore S requirements for entry under BSL2 conditions, S has been pseudotyped onto vesicular stomatitis virus (VSV) or retroviral particles with varied success. Several alterations to S were demonstrated to improve pseudoparticle titers, but they have not been systematically compared. In this study, we produced pseudotyped VSV particles with multiple modifications to S, including truncation, mutation, and tagging strategies. The main objective of this study was to determine which modifications of the S protein optimize cell surface expression, incorporation into pseudotyped particles, and pseudoparticle entry. Removal of the last 19 residues of the cytoplasmic tail produced a hyper-fusogenic S, while removal of 21 residues increased S surface production and VSV incorporation. Additionally, we engineered a replication-competent VSV (rVSV) virus to produce the S-D614G variant with a truncated cytoplasmic tail. While the particles can be used to assess S entry requirements, the rVSV∆G/S_Met1_D614G∆21 virus has a poor specific infectivity (particle to infectious titer ratio).

## 1. Introduction

The causative agent responsible for the global COVID-19 pandemic is the novel beta-coronavirus, severe acute respiratory syndrome coronavirus 2 (SARS-CoV-2). Due to their ability to mutate rapidly and undergo genetic recombination, coronaviruses are capable of adapting to new ecological niches and infecting a wide variety of animals including humans [1]. Previously known human coronaviruses cause mild respiratory illness, but recently, several highly pathogenic coronaviruses have emerged to cause severe respiratory disease and mortality in humans [2,3]. SARS-CoV-1 arose in China between 2002–2003 and quickly spread to several other countries, taking the lives of several hundred people [4]. A decade later, MERS-CoV emerged in the Middle East, resulting in over a thousand cases with a mortality rate approaching 35% [5]. In 2019, SARS-CoV-2 was identified in Wuhan, Hubei province, China and has since infected tens of millions of people across the globe, resulting in more than a million deaths [6]. The breadth and scale of the current COVID-19 pandemic are unprecedented. 

SARS-CoV-2 is an enveloped, positive-sense, single-stranded RNA virus with a ~30 kb genome that encodes four structural proteins: spike (S), membrane (M), envelope (E), and nucleocapsid (N) proteins [6,7]. For coronaviruses, the primary determinant of the host range and immunogenicity is the trimeric viral spike (S) glycoprotein [8]. In order to gain entry into a host cell, S initiates interaction with cellular receptors and mediates membrane fusion [8,9]. S is an ~180–200 kDa class I fusion protein that is post-translationally cleaved into S1 and S2 subunits that exist together in a metastable prefusion conformation on the surface of the virion [8,10]. The S1 receptor-binding domain (RBD) interacts with the host receptor angiotensin-converting enzyme 2 (ACE2), mediating viral attachment to a host cell [11,12]. The S2 subunit is composed of a short fusion peptide, two heptad repeat regions, and transmembrane domains, and is highly conserved across the coronavirus genera [13,14]. Proteolytic cleavage events, perhaps along with low pH and/or receptor binding, trigger conformational changes that allow the S2 fusion peptide to insert into the target membrane. Additional conformational changes result in the S2 heptad repeat regions (H1 and H2) forming a 6-helix bundle that merges viral and cellular membranes.

The proteolytic events that drive viral fusion can take place either at the plasma membrane (early pathway) or in endosomes (late pathway), depending on the proteases that are present within the host cell. When entering cells containing transmembrane protease serine subfamily member 2 (TMPRSS2), SARS-CoV-2 can fuse with the plasma membrane using the early pathway. TMPRSS2 is a protease found in lung cells that plays a key role in entry of several viruses, including influenza and SARS-CoV-1 [15,16,17]. Once S interacts with ACE2, TMPRSS2 cleaves S, facilitating membrane fusion at the plasma membrane [18]. For infection of cells lacking transmembrane proteases like TMPRSS2, SARS-CoV-2 enters using the late pathway. Viral particles bind to receptors, are internalized through endocytosis, and are trafficked to low pH endosomes [19]. The acidic environment activates endosomal cathepsin proteases [19]. Both cathepsin B in early endosomes and cathepsin L in late endosomes can mediate SARS-CoV-1 and -2 entry [17,20]. Once activated, these proteases cleave S, enabling fusion. Previous studies have shown SARS-CoV-1 more efficiently enters cells that produce TMPRSS2 over cathepsin [17,21].

In order to better understand the characteristics of SARS-CoV-2 S that determine cellular tropism and host range, it is critical to develop pseudotyped and recombinant systems that allow research to be conducted under BSL2 conditions. Use of authentic SARS-CoV-2 must be conducted in BSL3 containment facilities, which are not readily available at most institutions. Viral pseudo-typing is a strategy for overcoming biosafety level restrictions or difficulties in culturing viruses in vitro. Pseudotyped particles can be produced by coating the genetic core of one virus with the viral glycoprotein of interest studded in the lipid envelope. Vesicular stomatitis virus (VSV) is a popular pseudo-typing platform due to its ability to readily incorporate foreign glycoproteins into its budding progeny [22]. To generate pseudoparticles of VSV (ppVSV), cells producing the foreign glycoprotein are transduced with ppVSVΔG/G, which contain a genome lacking its glycoprotein (VSVΔG). The VSV machinery produces structural proteins that bud through the cellular plasma membrane and will incorporate foreign glycoproteins that are present. These newly produced ppVSV particles mimic the entry pathways of the viruses from which their glycoproteins are derived, but are not replication-competent since they lack genes encoding a glycoprotein and cannot produce infectious progeny [23,24,25]. Recombinant VSV (rVSV) particles are replication-competent viruses generated from altered VSV genomes in which the foreign glycoproteins have been cloned into the genome in place of VSV-G. Once recovered, these rVSV produce replication-competent progeny [26]. In order to produce both ppVSV and rVSV capable of entering cells with a foreign glycoprotein, the glycoprotein needs to be incorporated efficiently in a fusion active form. Modification of the desired surface glycoprotein may be required to ensure proper incorporation into the particle. For example, changes in the cytoplasmic tail that increase protein levels at the plasma membrane can increase particle production [27,28,29,30]. Both ppVSV and rVSV particles have been used as vaccine vectors including the FDA-approved Ervebo, an rVSV-ZEBOV [31,32]. 

Prior studies have demonstrated the ability of SARS-CoV-2 S to pseudotype using a variety of platforms, including human immunodeficiency virus 1 (HIV-1), murine leukemia virus (MLV), and VSV [14,17,33,34,35,36,37]. These studies utilize different means to achieve optimal pseudoparticle production, including varied truncation, mutation and tagging strategies for S. This study rigorously compares which modifications to S directly impact cell surface expression, incorporation into VSV pseudoparticles, and pseudoparticle entry.

## 2. Materials and Methods

### 2.1. Cells and Plasmids

Vero E6 (ATCC # CCL-81), BHK-21 (ATCC #CCL-10), BSR-T7/5 (BHK stably expressing T7 RNA polymerase) [38], and Vero-hSLAM [39] were maintained in Dulbecco’s Modified Eagle’s Medium (DMEM; Mediatech, Manassas, VA, USA) with 5% fetal bovine serum (FBS; Seradigm-VWR, Radnor, PA, USA). HEK293T cells (ATCC # CRL-11268) were maintained in DMEM with 10% FBS. Calu3 cells (ATCC HTB-55) were maintained in DMEM:F12 (Mediatech, Manassas, VA, USA) 10% FBS. All cells were maintained at 37°C with 5% CO_2_. BSR-T7/5 and Vero-hSLAM cells were passaged in G418 (5 mg/mL) once a week to maintain the T7 and hSLAM production respectively.

**Figure 1:**
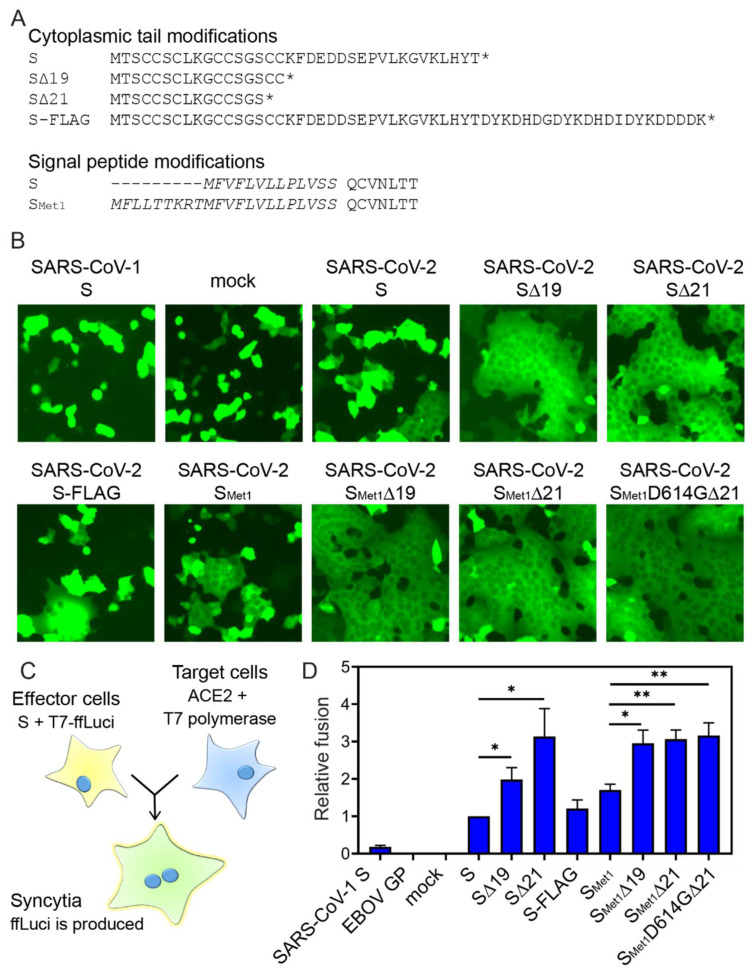
Cytoplasmic tail truncations increase fusion activity. (**A**) Protein sequences of the cytoplasmic tail modifications or signal peptide modifications. Residues shown in italics form the signal peptide. (**B**) SARS-CoV-2 induced syncytia. Vero-hSLAM cells were co-transfected with plasmids encoding the indicated viral fusion protein and pmaxGFP. Cell-to-cell fusion was assessed 24 hpi; magnification, ×20. Representative fields of view are shown. (**C**) Schematic representation of the cell-to-cell fusion assay. (**D**) Fusion was quantified by luminescence levels. Fusion efficiency was determined by comparing luciferase levels to the signal observed with full-length SARS-CoV-2 S protein. Fusion assays were completed in duplicate, three independent times. Data shown are the averages and standard error of the mean (SEM). *, *p* < 0.05; **, *p* < 0.01.

The SARS-CoV-2 Spike protein expression vector (pCAGG-SARS-CoV-2-codon optimized Spike) was generously provided by Dr. Biao He (UGA). We added restriction enzyme sites both upstream (MluI and KpnI) and downstream (XhoI and NheI) of the S open reading frame with PCR. In addition, primers were designed to remove the last 19 or 21 amino acids of the cytoplasmic tail and/or add a triple flag tag (Figure 1A). Primer sequences are available upon request. The PCR fragment was digested with KpnI and XhoI to clone the S ORF into pcDNA3.1intron backbone [40], or cut with MluI and NheI to ligate into the pVSV-ΔG-nLuciP-2.6, (adapted from pVSV-ΔG-GFP 2.6 courtesy of Michael Whitt; KeraFAST) [22]. Primers were designed to introduce the upstream signal peptide residues using In-Fusion cloning (Takara Bio, Mountain View, CA, USA). Plasmid alterations were confirmed by sequencing.

### 2.2. Syncytia Imaging

Vero-hSLAM cells were co-transfected with plasmids encoding the indicated viral fusion protein and pmaxGFP to readily observe syncytia formation. Syncytia were imaged twenty-four hours following transfection with the Zoe microscope (Bio-Rad, Hercules, CA, USA) (magnification, ×20).

### 2.3. Quantitative Cell-to-Cell Fusion Assay.

The quantitative cell-to-cell fusion assay was adapted from the measles fusion assay previously established [41]. Effector HEK293T cells were co-transfected with plasmids encoding the indicated virus fusion protein and a plasmid containing firefly luciferase under the control of a T7 promoter. Target HEK293T cells were transfected with a plasmid encoding for human ACE2. Twenty-four hours following transfection the target cells were infected with MVA-T7 to produce the T7 polymerase. Thirty-six hours following transfection, the target cells were washed with PBS, lifted, and overlaid onto the effector cells for five hours. Unfused target cells were gently washed away with PBS and the remaining cells were lysed in Steady-Glo (Promega, Madison, WI, USA) as per manufacturer’s instructions. Luminescence levels were measured in a GloMax Explorer Multimode Microplate Reader (Promega, Madison, WI, USA). Each S variant was assessed in the fusion assay in duplicate in four independent experiments. Fusion efficiency was compared to levels produced by the full-length SARS-CoV-2 S protein.

### 2.4. Surface Biotinylation

BHK cells were transfected with plasmids encoding the indicated viral fusion proteins. Thirty-six hours following transfection, the cells were washed with cold PBS and biotinylated with 0.5 mg/mL sulfosuccinimidyl-2-(bioinamido) ethyl-1,3-dithiopropionate (ThermoFisher, Waltham, MA, USA) for 30 min on ice. Following biotinylation, the reaction was quenched with DMEM 5% FBS for 10 min. Cells were washed three times in PBS and then lysed in 500 µL of M2 lysis buffer (50 mM Tris (pH 7.4), 150 mM NaCl, 1 mM EDTA, 1% Triton X-100) at 4 °C. Cell lysates were clarified via centrifugation (17,000× *g*, 10 min). Fifty microliters of each lysate were saved to examine protein levels in “total cell lysates”. To isolate proteins present on the surface of the plasma membrane, the remaining supernatant was incubated with streptavidin Sepharose beads (GE Healthcare, Chicago, IL, USA) for two hours at 4 °C while rotating. The beads were washed twice with Wash Buffer 1 (100 mM Tris (pH 7.6), 500 mM lithium chloride, 0.1% Triton X-100) and twice with Wash Buffer 2 (20 mM HEPES (pH 7.2), 2 mM EGTA, 10 mM magnesium chloride, 0.1% Triton X-100). Both the total lysate and surface fractions were incubated in urea buffer (200 mM Tris (pH 6.8), 8 M urea, 5% sodium dodecyl sulfate [SDS], 0.1 mM EDTA, 0.03% bromophenol blue, 1.5% dithiothreitol [DTT]) at 56 °C for 30 min and samples were subjected to immunoblot analysis. 

### 2.5. Antibodies and Immunoblots.

Surface biotinylated material and total cell lysates were fractionated by gel electrophoresis on 4-20% Tris-glycine gels (Invitrogen, Waltham, MA, USA) and transferred to polyvinylidene difluoride (PVDF) membranes (GE Healthcare, Chicago, IL, USA). The S2 spike subunit was detected with mouse anti-S2 monoclonal 1A9 (GeneTex, Irvine, CA, USA, cat#GTX632604); cellular actin was probed for with anti-actin monoclonal sc-47778 (Santa Cruz Biotechnology, Dallas, TX, USA). Each experiment was repeated at least three independent times, and representative data or images are shown in the figures. Trichloroacetic acid (TCA)-precipitated pseudotyped particles were fractionated as described for biotinylated material. Protein was detected with specific antibodies directed against S2 and against VSV matrix (23H12; courtesy of Douglas Lyles; Kerafast) [42]. Immunoblots were visualized using HRP-conjugated mouse IgG or rabbit IgG (Jackson ImmunoResearch, West Grove, PA, USA) secondary antibodies, and a ChemiDoc digital imaging system (Bio-Rad, Hercules, CA, USA). Immunoblot data were quantified using ImageLab software.

### 2.6. Generation of Pseudotyped Particles 

VSV pseudo-typed particles (ppVSV) were produced in HEK293T cells, as previously described [40,43]. Briefly, cells were transfected with the indicated viral glycoprotein expression vector. Twenty-four hours following transfection, the cells were transduced with either ppVSVΔG-GFP or ppVSVΔG-firefly pseudo-typed with VSV-G at a multiplicity of infection (MOI) of 1 for 1 h. The transduction media was removed, the cells were washed three times in PBS to remove any remaining particles, and fresh media was added. Newly formed ppVSV particles were collected 16- and 24-h following transduction, cleared of cellular debris, aliquoted, and frozen at −80 °C. 

### 2.7. Pseudotyped Entry Assay

Pseudotyped entry assays were completed as previously described [40,43]. Briefly, Vero or Calu3 cells were treated with equivalent volumes of pseudotyped particles and reporter gene production was assayed 16–24 h post addition. For ppVSVΔG-GFP detection, cells were trypsinised and fixed in 4% paraformaldehyde in PBS. GFP expression was analyzed by flow cytometry. For ppVSVΔG-ffLuc detection, firefly luciferase levels were assayed using the Steady-Glo luciferase assay system (Promega, Madison, WI, USA). 

### 2.8. S Incorporation Detection

VSV transduction particles were harvested and clarified by centrifugation (3000× *g*, 10 min). To monitor S incorporation, 1.5 mL of ppVSV particles were precipitated with 10% TCA for 30 min at 4°C. TCA-treated proteins were pelleted at (17,000× *g*, 30 min, 4°C), washed with acetone, and pelleted (17,000× *g*, 10 min, 4 °C). Pelleted TCA-treated proteins were dried and denatured in SDS-urea buffer at 95 °C for 10 min. Particles were subjected to immunoblot analysis for S2 and VSV matrix levels. 

### 2.9. Recovery of rVSV-CoV2-S

Replication-competent VSV encoding the SARS-CoV-2 Spike (S_Met1_D614G∆21) was recovered by transfecting BSR-T7 cells with plasmids encoding the full-length genome along with VSV N, P, and L (courtesy of Michael Whitt, University of Tennessee; Kerafast) [22]. Forty-eight hours following transfection the cells were overlaid onto Vero cells. Virus was amplified on Vero cells to produce pass 2 virus stock. RNA was isolated and sequenced to ensure the Spike sequence did not change during amplification.

### 2.10. Viral Replication Curves

Vero and Calu3 cells were plated at 2.5 × 10^5^ cells/mL. The following morning, cells were infected with either rVSV∆G-SARS-CoV2-S or rVSV-G at MOI 0.1. One hour following infection, inoculum was removed and fresh media was replaced. At the indicated time points supernatant was collected and media replaced. Supernatants were frozen at −80 °C and later titrated on Vero cells.

### 2.11. Live-Cell Luciferase Assay

Vero and Calu3 cells were plated in black-walled 96-well plates (40,000 cells per well). The following day, cell media was replaced with phenol red-free DMEM 5% FBS with 10 mM HEPES and Endurazine substrate (Promega, Madison, WI, USA). Cells were then incubated for 2 h to allow the cells to metabolize the substrate. Virus was then added to the cells (MOI 1), and some wells were treated with ammonium chloride (25 mM) at the time of infection. The plate was placed into a pre-warmed GloMax Explorer Multimode Microplate Reader (Promega, Madison, WI, USA) after infection. Luminescence was read every 10 min over the course of the experiment.

### 2.12. Specific Infectivity Determination

Specific infectivity was determined by comparing the number of viral genomes by the titer. RNA from viral stocks produced in Vero and Calu3 cells was isolated using the Viral RNA kit (Zymo Research, Irvine, CA, USA). RNA was converted to cDNA using the High Capacity RNA-to-cDNA kit (ThermoFisher, Waltham, MA, USA). Genome copies were quantified by qPCR using a DNA standard curve and a TaqMan probe that binds to the L region in the VSV genome. Stock titers were determined by serially diluting virus, adding to Vero cells, and monitoring cells for cytopathic effect. Tissue culture infectious dose 50 was determined by the Spearman-Karber method [44]. 

### 2.13. rVSV/SARS-CoV2 Antibody Neutralization Assay

rVSV∆G/SARS-CoV2-S_Met1_D614G∆21 virions (2500 PFU) were incubated with 4-fold dilutions of a neutralizing antibody (SARS-CoV-2 Spike neutralizing antibody, rabbit Mab, 40592-R001; Sino Biological; Beijing, China). The virus antibody mixture was incubated at room temperature for 30 min before the mixture was added to Vero cells (1.5 h, 37 °C). After infection, the inoculum was removed, cell media was replaced with phenol red-free DMEM 5% FBS containing 10 mM HEPES and Endurazine substrate (Promega, Madison, WI, USA), and the plate was placed into a pre-warmed GloMax Explorer Multimode Microplate Reader (Promega, Madison, WI, USA). Luminescence was read every 10 min over the course of the experiment.

### 2.14. rVSV/SARS-CoV2-S Entry and Cell Viability Assays with Inhibitors

Vero and Calu3 cells were pre-treated 2 h prior to infection with inhibitors: ammonium chloride (25 mM), bafilomycin A1 (250 nM), camostat mesylate (50 µM), E64d (50 µM), or solvent (DMSO or Ethanol) (all inhibitors from Millipore Sigma, Burlington, MA, USA). For Vero cells, rVSV∆G/SARS-CoV2-S_Met1_D614G∆21 was added at an MOI of 0.5 and nano-luciferase production was detected 5 h post-infection. For Calu3 cells, rVSV∆G/SARS-CoV2-S_Met1_D614G∆21 was added at an MOI of 0.1 and nano-luciferase levels were detected 16 h post-infection. Nano-luciferase was quantified using the Nano-Glo luciferase assay system (Promega, Madison, WI, USA). Cell viability was monitored using CellTiter-Glo (Promega, Madison, WI, USA) in both cell lines after treatment with the inhibitors and measured when nano-luciferase was examined. 

## 3. Results

### 3.1. Trimming of the SARS-CoV-2 S Cytoplasmic Tail Enhances Cell-Cell Fusion.

Full-length codon optimized SARS-CoV-2 S ORF was introduced into the pcDNA3.1intron vector. We produced nine S constructs and examined fusogenicity, surface expression, and production and transduction efficiency of VSV pseudotyped particles. First, we added a 3×FLAG tag to the C-terminus of full-length S. Prior reports have successfully pseudotyped S with 3×FLAG or HA tag incorporation at the C-terminus [14,17]. We also created a series of S constructs where the C-terminal cytoplasmic tail was truncated, removing the last 19 or 21 amino acids (Figure 1A). Deleting the last 19 amino acids was previously shown to increase pseudo-typing efficiency [14,34,37], and two independent groups rescued replication-competent VSV SARS-CoV-2 S chimeric viruses that lacked the last 21 amino acid residues of the cytoplasmic tail [45,46]. In addition, the S ORF was altered to reflect a previously proposed upstream in-frame alternate start codon (referred to with the subscript Met1) that alters the signal peptidase by adding an additional nine amino acids to the N-terminus (Figure 1A) [47]. Lastly, the S_Met1_D614G∆21 construct was implemented because the emergence of this prominent SNP (D614G) during the SARS-CoV-2 pandemic has been associated with increased viral load and disease severity in infected individuals [48]. It has been suggested that the D614G mutation alters the conformation of S such that it more readily binds hACE2 [49]. SARS-CoV-2 variants containing the D614G mutation have been shown to promote enhanced pseudotyped lentiviral particle infectivity and increased VSV pseudotype efficiency [37,49]. 

In order to determine whether the S variants differed in their ability to fuse at the plasma membrane, the panel of S constructs was co-transfected with a GFP reporter plasmid into Vero cells and fusion activity was observed 24 h post-transfection (Figure 1B). Full-length SARS-CoV-1 S and GFP alone were included as controls. Truncation of the SARS-CoV-1 S cytoplasmic tail was previously shown to be essential for efficient pseudo-typing [50,51]. SARS-CoV-1 S also requires exogenous trypsin or protease cleavage to activate cell-cell fusion. Thus the lack of syncytia (multi-nucleated cells) formation in SARS-CoV-1 transfected cells was not surprising [52]. While cells producing full-length SARS-CoV-2 S formed small syncytia, all the cytoplasmic tail deletions caused robust cell-cell fusion regardless of start codon usage (Figure 1B). 

To quantify the level of cell-to-cell fusion, we adapted a fusion assay for SARS-CoV-2 S (Figure 1C). Effector cells were transfected with the SARS-CoV-2 S constructs as well as a plasmid encoding a firefly reporter gene driven by the T7 polymerase. Target cells were transfected with a plasmid encoding human ACE2 and were infected with MVA-T7, which produces the T7 RNA polymerase. The effector and target cells were mixed for five hours to allow S-mediated fusion. Cell-to-cell fusion enabled the T7 polymerase to produce firefly luciferase, and luciferase levels were quantified and compared to the full-length SARS-CoV-2 S (Figure 1D). We did not observe a significant difference in full-length S versus S-FLAG cell-cell fusion. Removing the last 19 or 21 residues of the cytoplasmic tail significantly increased fusion over the full-length S, but fusion was not significantly different between S∆19 and S∆21 truncations. Similar trends were observed in the constructs containing the longer signal peptide. Full-length S_Met1_ showed significantly more fusion activity compared to the S construct (*p* = 0.0205). While the additional residues in the signal peptide increased full-length S fusion, no increase in fusion was observed between S∆19 and S_Met1_∆19, as previously shown [47], or between S∆21 and S_Met1_∆21. The D614G variant did not further enhance the fusion activity of the S∆21 construct. These data confirm that S cytoplasmic tail truncations most effectively enhanced cell-to-cell fusion. 

### 3.2. Truncations in the Cytoplasmic Tail Do Not Necessarily Increase S Protein Levels on the Cell Surface.

**Figure 2:**
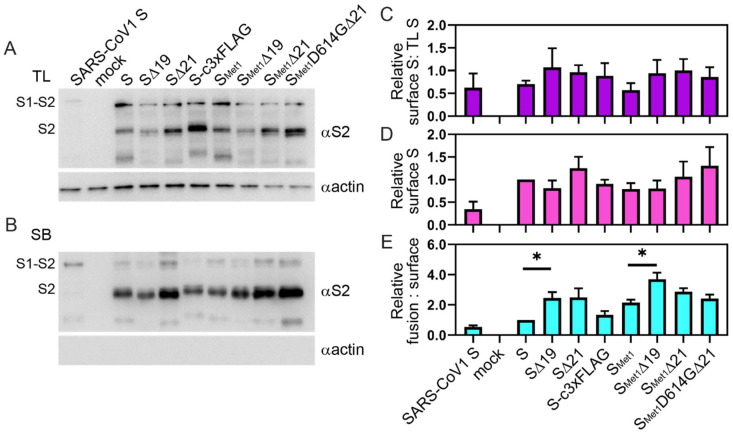
Surface levels of S variants. BHK cells were transfected with plasmids encoding the indicated viral protein or negative control. After 36 h, cells were subjected to surface biotinylation. Precipitated proteins were separated via SDS-PAGE. Immunoblot assays were performed to detect levels of S2 expression in total cell lysates (**A**) and biotinylated surface material (**B**) with an anti-S2 antibody. Immunoblots were also probed for actin as a loading control. The immunoblot shown is representative of four independent trials. Immunoblots were quantified the ratio of surface S2 was compared to the level of S2 present in the total lysates (**C**), and the level of variant S on the surface was compared to the level of full-length S (**D**). The level of surface-produced S was also compared to the relative fusion activity shown in Figure 1 to determine the relative fusion activity to S protein level (**E**). Data shown are the averages and SEM. *, *p* < 0.05.

S-induced syncytia formation can only be mediated by S protein present on the surface of cells. Coronaviruses are known to bud from internal cellular membranes, and therefore contain ER retention signals in the S cytoplasmic tail that retain S in the internal membrane for virus assembly. To determine if the cytoplasmic tail truncations increase the protein levels of S on the plasma membrane, we compared the levels of S within the total cell lysates (TL) to those present on the surface using surface biotinylation. We observed a slight, but not significant, trend indicating that more S was able to reach the surface when the cytoplasmic tail was truncated (Figure 2A–C). While not significant, the S∆19 variants displayed either similar or lower surface expression relative to full-length S, while S∆21 surface levels were slightly increased (Figure 2D). When comparing fusion activity with S variant surface levels, the S∆19 constructs produced significantly more fusion for the level of S found on the plasma membrane than the full-length S constructs, suggesting that truncating the last 19 residues of the cytoplasmic tail may make a hyper-fusogenic S (Figure 2E). Thus, cytoplasmic tail truncations only slightly impact cell surface S production, however, specifically removing the last 19 residues improves surface S fusion efficiency. 

### 3.3. S Cytoplasmic Tail Deletions Enhance ppVSV Transduction Efficiency

**Figure 3:**
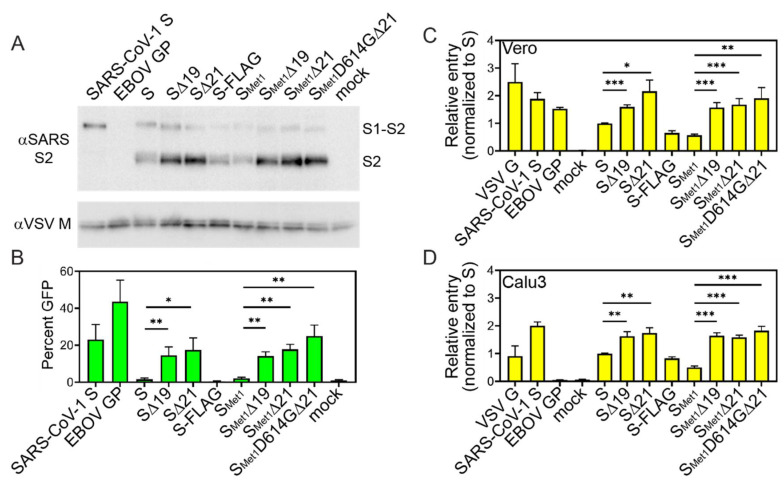
**S** variants incorporation in ppVSV and transduction efficiency. ppVSV particles were produced with the indicated viral glycoprotein. Particles were concentrated and analyzed for glycoprotein incorporation by separating proteins by SDS-PAGE followed by immunoblot analysis for both VSV M and the S2 subunit of SARS-CoV-2 (**A**). Particles were produced and analyzed three independent times; representative immunoblots are shown. (**B**) Equal volumes of ppVSV-GFP coated in the indicated viral glycoprotein were added to Vero cells. GFP positive cells were enumerated 16 h post-transduction with a flow cytometer. Data represent the percentage of Vero cells that produced the GFP reporter (**B**). Equal volumes of ppVSV-ffLuc coated in the indicated viral glycoprotein were added to Vero cells (**C**) or Calu3 cells (**D**) and luciferase levels were quantified 16 h following infection. Data represent the luciferase signal relative to full-length SARS-CoV-2 S levels. All transduction experiments were assessed in three independent experiments. Data shown are the averages and SEM. *, *p* < 0.05; **, *p* < 0.01; ***, *p* < 0.001.

VSV pseudo-particles coated in the different S constructs were generated in 293T cells and examined for VSV-S incorporation and transduction efficiencies. S constructs containing cytoplasmic tail deletions were more readily incorporated into pseudo-particles (Figure 3A). To determine if the particles can mediate cell entry, we monitored cells for reporter gene expression following transduction. Equal volumes of ppVSV-GFP were added to Vero cells and the number of GFP positive cells was quantified 16 h post-transduction. In general, the transduction efficiency of S ppVSV was low, with full-length S producing only slightly more positive green cells than the no envelope (mock) control (Figure 3B). Deletion of the cytoplasmic tail significantly increased transduction above full-length S, but there were no significant differences among the truncated S constructs (Figure 3B). Similar results were observed when ppVSV-ffLuc (firefly luciferase) particles were produced. S-ppVSV-ffLuc entry into both Vero and Calu3, a human lung cell line, produced a similar pattern (Figure 3C,D). Surprisingly, the VSV G control poorly entered Calu3 cells, as did EBOV GP which was previously observed (Figure 3D) [53]. 

### 3.4. rVSV/S_Met1_D614G∆21 Recombinant Virus Produces Relatively Low Titers with Poor Specific Infectivity

**Figure 4:**
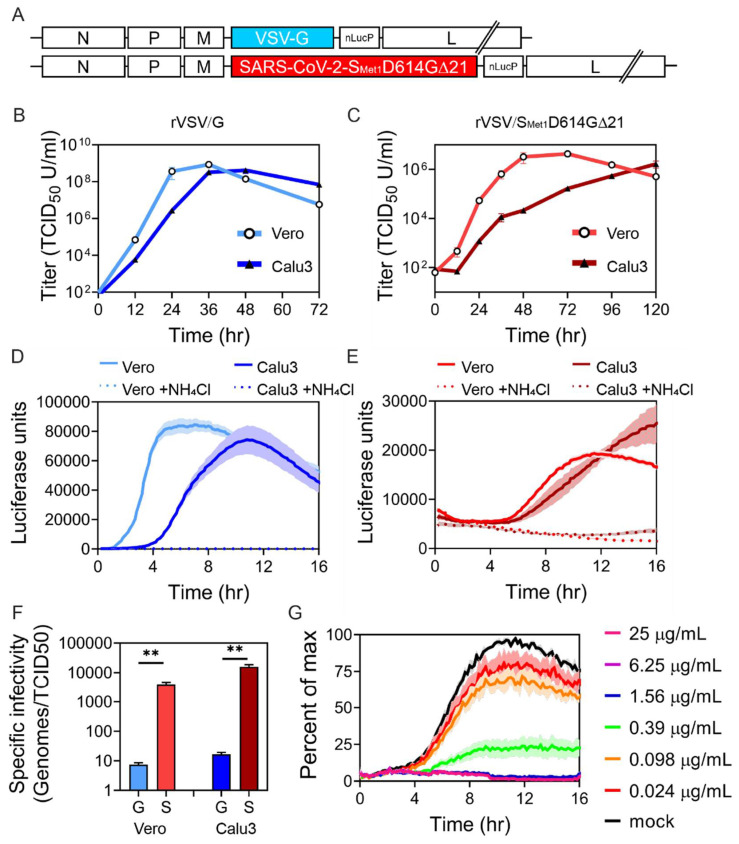
rVSV-SARS-CoV-2-S_Met1_D614G∆21 replicates slowly but can be used to characterize S dependent entry. (**A**) Schematic of the engineered genome containing either the VSV-G or the SARS-CoV-2-S_Met1_D614G∆21. Both include the nano-luciferase-P reporter gene after the glycoprotein. Multi-cycle replication curves (MOI 0.01) in both Vero and Calu3 cells using rVSV/G (**B**) or rVSV/S_Met1_D614G∆21 (**C**). Luciferase levels were monitored in live cells after infecting cells with rVSV/G (**D**) or rVSV/S_Met1_D614G∆21 (**E**). In the indicated samples, ammonium chloride (NH_4_Cl) was added at the time of infection to establish the background within the assay. (**F**) Specific infectivity of viral particles was quantified by comparing the number of viral genomes within the sample to the infectious titers. rVSV/G samples are labeled G and rVSV/S_Met1_D614G∆21 samples are labeled S. (**G**) Real-time neutralization assay. rVSV/S_Met1_D614G∆21 was pre-incubated with decreasing concentrations of a neutralizing antibody (40592-R001, Sino Biological). The virus antibody mixture was added to Vero cells for 1.5 h at 37 °C. The inoculum was then removed, and luminescence was measured every 10 min over the course of the experiment. Data shown are the averages and SEM of three independent experiments performed in duplicate. **, *p* < 0.01.

Although pseudo-particles are useful for some assays, replication-competent particles enable a wider array of experimental questions to be addressed. Therefore, S_Met1_D614G∆21 was cloned into the VSV genome in place of the glycoprotein to produce a replication-competent virus (Figure 4A). A nano-luciferase-PEST (nLucP) reporter was also added to the genome to monitor replication. Virus was recovered by transfecting the molecular clone and helper plasmids into BSR cells that were overlaid with Vero cells. The produced virus was amplified by passing the virus onto Vero cells one time. Virus replication was characterized in both Vero and Calu3 cells after a low MOI infection (0.01) (Figure 4B,C). rVSV/G growth was slightly delayed in Calu3 cells compared to Vero cells, but we observed high viral titers in both cell lines 36–48 h following infection, after which time the cell monolayer exhibited robust cytopathic effect and cell death (Figure 4B). rVSV/S_Met1_D614G∆21 spread through both Vero and Calu3 at a much slower rate, reaching peak titers in Vero cells between 48–72 h and 5 days in Calu3 cells (Figure 4C). While rVSV/G replication peaked at greater than 10^8^ TCID_50_ U/mL, rVSV/S_Met1_D614G∆21 peak titer was 100 times lower.

We also monitored reporter gene production over time in live cells. Virus was added to either Vero or Calu3 cells and luciferase activity was measured every ten minutes (Figure 4D,E). Some cells were infected with virus and simultaneously treated with ammonium chloride (NH_4_Cl) to inhibit low pH mediated entry. Again, we observed rapid and robust rVSV/G replication in Vero cells, which was slightly delayed in Calu3 cells (Figure 4D). rVSV/S_Met1_D614G∆21 entry was delayed and luciferase activity remained low, suggesting only the first round of infection was detected in the 16 h time frame (Figure 4E). We also consistently observed a higher background signal in the rVSV/S_Met1_D614G∆21 infection due to the high volume of inoculum needed for the experiment, suggesting that the individual particle infectivity rate may be low. To determine the specific infectivity, or the number of viral particles required to infect a cell, we quantified the number of genomes present in the viral stock and compared them to the infectious titer (Figure 4F). rVSV/G particles were very infectious, with approximately 10 particles required to produce infection. In contrast, rVSV/S_Met1_D614G∆21 particles required between 1000 and 10,000 genomes to productively infect, suggesting that although the recombinant virus is capable of infection, either the incorporation, stability or fusion activity of S is inefficient.

As a proof of concept experiment, we pre-incubated the rVSV/S_Met1_D614G∆21 with decreasing concentrations of an established neutralizing antibody. The antibody/virus mixture was then added to Vero cells and luciferase activity was monitored in the live-cell assay (Figure 4G). The highest concentrations of antibody completely blocked luciferase production. We calculated an IC50 of 0.24 µg/mL for the antibody, which is similar to that found by Sino Biological who supplied the antibody.

### 3.5. rVSV/S_Met1_D614G∆21 Can Be Used to Characterize Cellular Requirements for Entry

**Figure 5:**
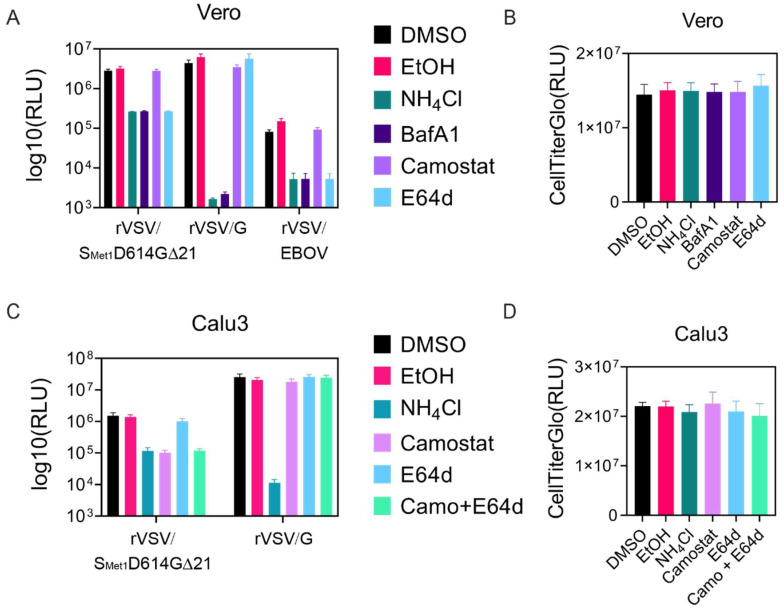
rVSV-SARS-CoV-2-S_Met1_D614G∆21 entry is sensitive to protease inhibitors in a cell-specific manner. (**A**) Vero cells were infected with rVSV/S_Met1_D614G∆21, rVSV/G, or rVSV/EBOV-GP (MOI 0.01) in the presence of the indicated compounds. Luciferase was quantified 5 h following infection. (**B**) Vero cell viability measured at 18 h post treatment. (**C**) Calu3 cells were infected with rVSV/S_Met1_D614G∆21 or rVSV/G (MOI 0.01) in the presence of the indicated compounds. Luciferase was quantified 16 h following infection. (**D**) Calu3 cell viability measured at 18 h post treatment. Each experiment was repeated in triplicate, three independent times. Data shown are the averages and SEM.

SARS-CoV-2 S-mediated entry into host cells can occur at the plasma membrane or within endosomes and relies on the ACE2 receptor, as well as proteolytic priming by TMPRSS2 and/or lysosomal cathepsin proteases [17]. rVSV/S_Met1_D614G∆21 entry assays were performed in Vero and Calu3 cells to determine which pathways might be utilized. While both Vero and Calu3 cells express the ACE2 receptor, Vero cells lack TMPRSS2 and Calu3 cells express very low levels of CatL [17,53]. We included rVSV/G in both cell lines as a control, as VSV G relies on pH-dependent endosomal fusion without proteolytic activation. rVSV/EBOV was included as an additional control for entry into Vero cells since EBOV GP fusion relies on cathepsin mediated cleavage and low pH [54]. Treatment with ammonium chloride and bafilomycin A1 raises the pH within endosomes, which blocks cathepsin activation as well as pH-dependent fusion. Both ammonium chloride and bafilomycin A1 blocked entry of all three viruses into Vero cells (Figure 5A) without altering cell viability (Figure 5B). Vero cells lack TMPRRS2 but contain cathepsins, suggesting that SARS-CoV-2 S mediated entry should occur through the late pathway and be sensitive to cathepsin inhibitors such as E64d. As expected, camo-stat, a TMPRRS2 inhibitor, did not affect rVSV/S_Met1_D614G∆21 entry into Vero cells, but E64d inhibited entry approximately 90%. rVSV/EBOV entry was also blocked by E64d whereas rVSV/G entry was unaffected (Figure 5A).

Calu3 cells produce TMPRRS2 on the cell surface and therefore rVSV/S_Met1_D614G∆21 entry should occur through the early pathway. rVSV/S_Met1_D614G∆21 entry into TMPRSS2 positive Calu3 cells was significantly inhibited by camo-stat treatment (Figure 5C), while treatment with cathepsin inhibitor E64d had no effect (Figure 5C). We did not detect a synergistic effect by adding both camo-stat and E64d together. The drug treatments did not affect cell viability (Figure 5D). In summary, these results support the current model of multi-mode entry of SARS-CoV-2 S and demonstrate that rVSV/S_Met1_D614G∆21 is capable of undergoing either TMPRSS2 and/or cathepsin mediated proteolytic priming and activation.

## 4. Discussion

This work compares various modifications to the SARS-CoV-2 Spike (S) with the goal of identifying the optimal S for VSV pseudoparticle production. These S-incorporating VSV pseudoparticles can ultimately serve as a tool to screen entry inhibitors and better define SARS-CoV-2 entry and tropism characteristics. The S variants generated combine varied truncation, mutation and tagging strategies (Figure 1A). Although several groups have successfully utilized some of these S variants to generate VSV pseudoparticles, this is the first study to compare how S modifications impact cell surface expression, incorporation into VSV pseudoparticles, and pseudoparticle transduction. The S variant that mediated transduction most efficiently was further utilized to generate replication-competent VSV, rVSV∆G/S_Met1_D614G∆21. This recombinant virus exhibited lower specific infectivity and reduced replication in comparison to rVSV/G. However, this model could be used to study the entry characteristics of SARS-CoV-2 (Figure 4 and Figure 5). 

The addition of a 3×FLAG tag to the C-terminus of S did not render any significant alterations in S; tagged and untagged S performed similarly in fusion, surface expression, and pseudoparticle transduction efficiency assays. We have previously observed a significant decrease in ppVSV titers when adding a 3×FLAG tag to the cytoplasmic tail of the LASV glycoprotein without altering fusion activity in a cell-to-cell fusion assay [40]. While LASV GP produces significantly higher ppVSV titers than SARS-CoV-2 S, these observations suggest 3×FLAG tags do not always reduce transduction efficiency and individual glycoproteins need to be evaluated for the impact of specific modifications on ppVSV incorporation and infectivity.

Based on signal peptide prediction algorithms and protein sequence alignments, it appears that the early annotated genome of SARS-CoV-2 missed nine residues that should be included in the S open reading frame [47]. Codon optimized constructs designed to produce the original S sequence therefore contained this shorter signal peptide that was predicted to be poorly recognized. We expected addition of the nine upstream residues would produce a signal peptide more favorable to recognition by Sec61 and increase protein levels. When the S start codon was altered to utilize an alternate upstream in-frame methionine, full-length S_Met1_ showed significantly more fusion activity compared to the S construct, although the increase was modest. However, immunoblot analysis did not suggest the protein levels were significantly increased and similarly S_Met1_ did not lead to enhanced incorporation into pseudo-particles or ppVSV transduction. Therefore, both open reading frames produce similar levels of S protein.

In order to efficiently pseudotype heterologous glycoproteins onto lentiviral, retroviral, and VSV based systems, changes to the cytoplasmic tail may be necessary [50,55,56,57]. These changes can alter cellular localization, producing more glycoprotein at sites of viral budding. Alternatively, changes to the cytoplasmic tail may alter fusion activation in a foreign system. This study compared different truncations to the cytoplasmic tail of S, including removal of 19 or 21 amino acids. Deleting 19 amino acids was previously shown to increase pseudo-typing efficiency of SARS-CoV-2 S [14,34,37], while rescuing and passaging of rVSV/SARS-CoV-2 viruses resulted in a 21 amino acid deletion, suggesting the cytoplasmic tail impedes recovery of rVSV/SARS-CoV-2 [45,46]. We chose to compare these two cytoplasmic tail deletions since amino acids 20 and 21 are cysteine residues, deletion of which could have important structural implications. 

While both cytoplasmic tail truncations remove the KxHxx ER retention signal, introducing point mutations into the retention motif was previously shown to be ineffective at boosting pseudoparticle production, suggesting that the length of the cytoplasmic tail, rather than the ER retention signal, is important for virus propagation using the rVSV system [36]. Consistent with these results, the ∆19 and ∆21 truncations did not significantly enhance cell surface production (Figure 2), but both modifications did enhance cell-to-cell fusion (Figure 1) and ppVSV titers (Figure 3). There were no significant differences between the ∆19 and ∆21 truncations during the fusion, surface expression or ppVSV production assays. Enhanced pseudoparticle production and infectivity following cytoplasmic tail deletion is generally associated with enhanced packaging efficiency or biological activity. The results here indicate that truncation of SARS-CoV-2 cytoplasmic tail may enhance pseudoparticle production and transduction efficiency by increasing S incorporation into particles and/or heightening fusogenic properties of S. We attempted to recover rVSV∆G/S∆19 with the various modifications tested, but were unsuccessful. Importantly, previous work comparing neutralization profiles of a clinical isolate of SARS-CoV-2 with a recombinant VSV virus incorporating a S∆21 has demonstrated that cytoplasmic tail truncation by 21 amino acids does not alter the Spike ectodomain neutralization profile [45], suggesting the more efficiently produced ppVSV/S particles can be used as a BSL2 alternative in assessing neutralization activities. 

As SARS-CoV-2 spreads around the globe, virus sequence analysis has identified S-G614 as the most prevalent S variant currently circulating, suggesting it may have a selective advantage [48]. In various pseudotyped particle and viral particle assays, there appears to be little if any increase in viral titers with S-G614 in tissue culture settings [37,58,59,60]. Here, the addition of D614G to S_Met1_∆21 did not significantly enhance S incorporation onto pseudoparticles or transduction efficiency beyond the enhancement from truncating the cytoplasmic tail. However, since the D614G mutation predominates in current circulating strains, recombinant VSV was generated to incorporate the S_Met1_D614G∆21 variant. rVSV/S_Met1_D614G∆21 was recovered, but particles produced during infection of both Vero and Calu3 cells exhibited low specific infectivity. All experiments were performed with virus that was minimally passaged, and sequence confirmed no additional changes to the S protein. Other rVSV∆G/SARS-CoV-2-S studies suggest passing the virus in cell culture results in adaptation to the system and produces higher titers [45,46]. This indicates that the incorporation, stability, or function of S_Met1_D614G∆21 S may be suboptimal in the replication-competent VSV system and additional selection in cell culture may produce an S variant that would produce higher titers. While inefficient, the rVSV/S_Met1_D614G∆21 that is produced can be an effective tool to characterize SARS-CoV-2 entry. The rVSV/S_Met1_D614G∆21 encoded nano-luciferase reporter production is specifically blocked by both neutralizing antibodies and established SARS-CoV-2 entry inhibitors that can be utilized under BSL2 containment to better understand SARS-CoV-2 entry and screen for therapeutics.

## 5. Conclusions

Establishing a BSL2 model for SARS-CoV-2 replication is critical for rapidly progressing our understanding of this virus and managing the ongoing pandemic, as these models exponentially increase the number of facilities and researchers that can contribute. Here we characterized SARS-CoV-2 S variants used in recent SARS-CoV-2 studies via cell-cell fusion and cell surface production assays and examined their abilities to mediate VSV pseudoparticle transduction. We found that full-length S can tolerate a C terminal 3×FLAG tag, which broadens the number of strategies researchers can use to biochemically detect full-length S in a laboratory setting. We additionally observed that S cytoplasmic tail truncations can improve yields of functional S-coated VSV pseudoparticles, which can enhance the production of these particles for inhibitor screens, neutralization assays, entry assays, and other experiments where single-cycle virions are appropriate. Finally, we generated a replication-competent recombinant rVSV virus containing S_Met1_D614G∆21 that can be used in the aforementioned assays and multi-cycle viral replication experiments; however, further optimization may be required if greater levels of viral production are desired.
